# *In situ* measurements of micronutrient dynamics in open seawater show that complex dissociation rates may limit diatom growth

**DOI:** 10.1038/s41598-018-34465-w

**Published:** 2018-10-31

**Authors:** Willy Baeyens, Yue Gao, William Davison, Josep Galceran, Martine Leermakers, Jaume Puy, Pierre-Jean Superville, Laurent Beguery

**Affiliations:** 1 Vrije Universiteit Brussel, Analytical, Environmental and Geochemistry Department (AMGC), 1050 Brussels, Belgium; 2 University of Lancaster, Lancaster Environment Centre, Lancaster, LA1 4YW UK; 3 University of Lleida, Departament de Quimica, 25198 Lleida, Spain; 4 Université de Lille, Laboratoire de Spectrochimie Infrarouge et Raman LASIR, CNRS, UMR 8516, 59000 Lille, France; 5 ALSEAMAR, 13590 Meyreuil, France

## Abstract

In this first *in situ* study of the dynamic availability of phytoplankton micronutrients, a SeaExplorer glider was combined with Diffusive Gradients in Thin Films and deployed in the Mediterranean Sea. On the basis of their labile metal complex pools, we discovered that Fe and Co can be potentially limiting and Cu co-limiting to diatom growth, contrary to the generally accepted view that phosphorus (phosphate) is the growth limiting element in the Mediterranean Sea. For flagellates and picoplankton, phosphorus remains the main element limiting growth. Our *in situ* measurements showed that organic complexes of Fe and Cu (>98% of total dissolved concentration), dissociate slower than inorganic complexes of Co, Cd and Ni (>99% of total dissolved concentration being free ions and inorganic complexes). This strengthens the potential growth limiting effect of Fe and Cu versus phosphate, which is present as a free ion and, thus, directly available for plankton.

## Introduction

In the contemporary ocean, photosynthetic carbon fixation by marine phytoplankton leads to the formation of ~45 gigatons of organic carbon (C) per annum, of which ~11 gigatons are exported to the ocean interior^1^. To sustain this C flux through marine ecosystems, nutrients - not only the major ones such as carbon, nitrogen or phosphate, but also micronutrients such as, for example, iron, manganese or copper^2^-must be sufficiently available to supply the required elemental composition of marine phytoplankton species. Phytoplankton need micronutrients because they play an important role in their metabolism: for example, Co, Cd and Zn in carbon dioxide acquisition; Fe and Mn in carbon fixation; Zn, Cd and Se in silica uptake; Fe and Mo in N_2_ fixation and Fe, Cu and Ni in organic N utilization^3^. Although most attention has been focused on Fe exerting a global limitation on primary productivity *e.g*.^4–6^, many of the other trace elements are also close to their potentially limiting values^7^. In prior studies of the Peru upwelling region, Co scarcity and speciation was found to influence phytoplankton species composition^8^ Fe, Zn and Co were mediating phytoplankton community structure in the Southern Ocean^9^ and Fe, Mn, Cu and Zn were found to be co-limiting productivity in the subarctic Pacific Ocean^10^.

In addition to the free hydrated ions, essential trace metals in seawater are present in different chemical forms since they interact with simple inorganic ligands, organic macromolecules, colloids (nominally >1000 kDa to <0.2 μm) and particles (>0.2 μm). Some of these complexes are highly labile indicating that dissociation is fast enough in comparison with diffusion to reach equilibrium with the free metal in relevant spatial and time domains. Other complexes with slower dissociation rates are partially labile or inert. Availability depends generally on both mobility (diffusivity when diffusion is the only transport phenomena) and lability (which indicates the ability of complexes to dissociate and contribute to the metal flux). Colloidal forms are predominantly inaccessible to phytoplankton^3,11^. While it is well established^12^ that complexation generally limits the availability of metals, microorganisms may also have receptors that target entire metal-ligand complexes, which is not dependent on dissociation of the complex before uptake, such as Ton B dependent receptors^13,14^.

Labile metal concentrations can be assessed with the Diffusive Gradient in Thin Films technique (DGT)^15^. The classic DGT device for trace metals uses a diffusive gel layer (controlling the mass transport) overlying a Chelex-100 resin gel layer (acting as a sink for metals). By dissociation, complexes contribute to the flux in both DGT and ASV, but to markedly different extents. The effective measurement time (which is the time metal complexes take to diffuse before they dissociate and accumulate on the electrode or resin) is higher for DGT than for ASV^16^. Thus, the two techniques can provide complementary information. A much wider range of metals can be measured by DGT compared to Cd, Cu, Pb and Zn usually measured by ASV. DGT devices with different thicknesses of the diffusion domain have been used for the *in situ* assessment of the lability of metal complexes^16,17^.

In most areas of the oceans, total dissolved concentrations of essential trace metals are very low, in the nanomolar to sub-nanomolar range^15,18,19^. For determination of their labile fractions techniques need to be more sensitive and selective: ideally capable of distinguishing between inert, partially labile and labile metal complexes^15,20,21^.

Here we present and discuss labile trace metal concentrations in the Mediterranean Sea, which is generally considered to be an oligotrophic basin with some local regions of enhanced productivity^22^. Total dissolved trace metal concentrations are generally low^18,23^, but there are no labile trace metal data. Our goal was to quantify and characterize labile metal complexes using a novel *in situ* approach that avoided possible sampling and sample treatment artefacts, including redistribution of complex species^24–26^.

**Figure 1:**
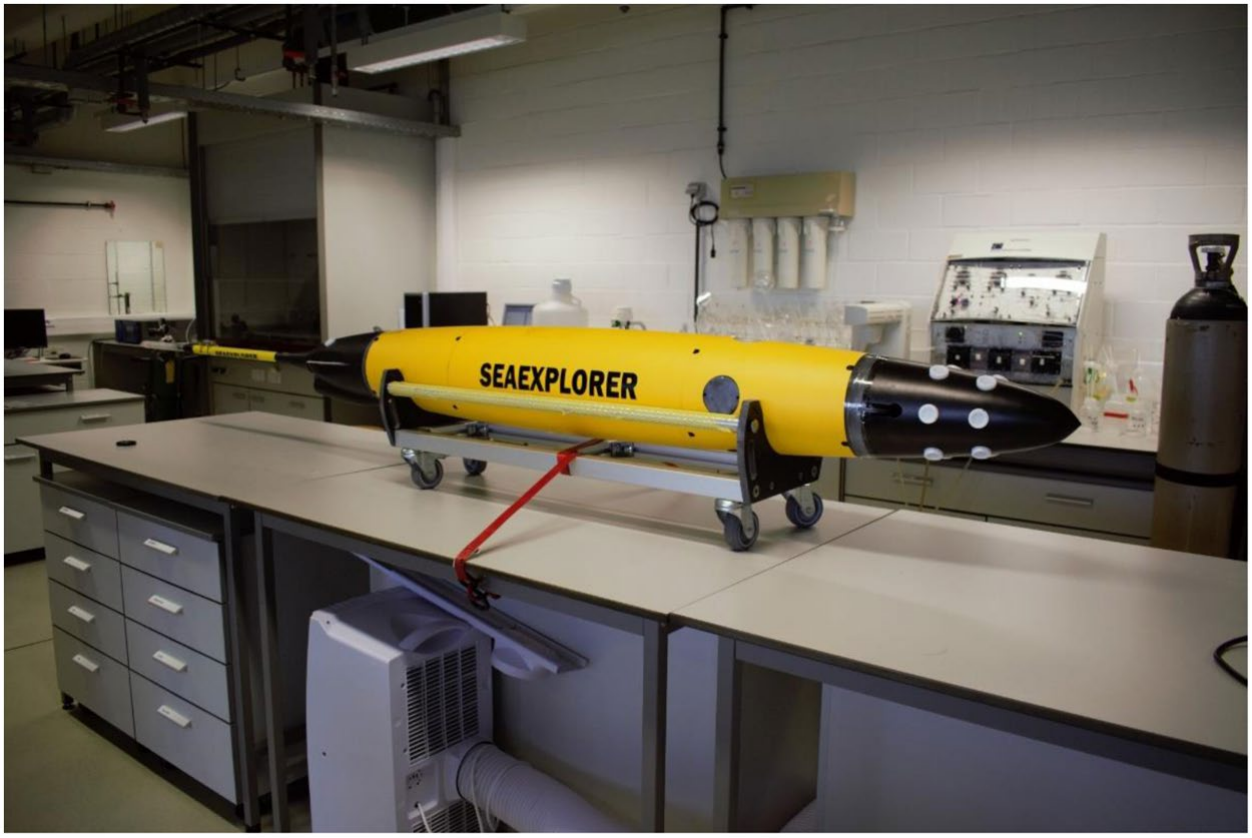
View of the modified SeaExplorer glider.

To realize our objective, we modified and used an autonomous underwater glider (SeaExplorer, Alseamar, France), equipping it with DGT samplers while eliminating potential self-contamination (Fig. 1). With this glider, a two-way cruise of 3 weeks was carried out between Isle du Levant (Hyères, France) and Corsica in the Mediterranean Sea (Fig. S1). Fast DGTs (diffusive domain of 0.0325 cm), measuring free ions and fast dissociating complexes and classic DGTs (diffusive domain of 0.1125 cm), measuring in addition slower dissociating complexes, were mounted on the SeaExplorer. Lehto *et al*.^27^ used an even thinner diffusive domain (0.0081 cm) than we did for our fast DGT, but the thin Nuclepore membrane involved (0.001 cm) was only used in laboratory conditions and never in the field.

Labile trace metal data and cellular metal quotas in plankton averaged over a time scale of a month will be used to estimate potential plankton growth limitation in the Mediterranean Sea. Such information was not previously available. In addition, average lability and dissociation rate of each sub-pool of the total dissolved trace metal concentration (free metal, inorganic and organic complexed metal) will be calculated using a new model.

## Results

The reliability of determinations of ultra-trace labile metal concentration in the oceans depends on several factors, the most important being adequate sensitivity and minimization of contamination during the whole analytical process. Both issues have been rigorously considered in this study. Sensitivity is always challenging when assessing concentrations at the pM level, but the in-built pre-concentration features of DGT overcome these difficulties. The *in situ* deployment of DGT avoids, thereby, many potential contamination problems.

In the Mediterranean Sea campaign, we assessed highly labile (3 fast DGTs at the head of the glider) and partially labile (5 classic DGTs at the head and 2 at the tail of the glider) metal complexes. The accumulation differences between devices with thick and thin diffusion domains are due to the residence time of the complexes in the diffusive domain. In the Mediterranean Sea experiment, the residence time of the metal complexes in the thick diffusive domain is more than 10 times longer than in the thin one, computed as the square of the ratio of diffusion domain thicknesses.

**Figure 2:**
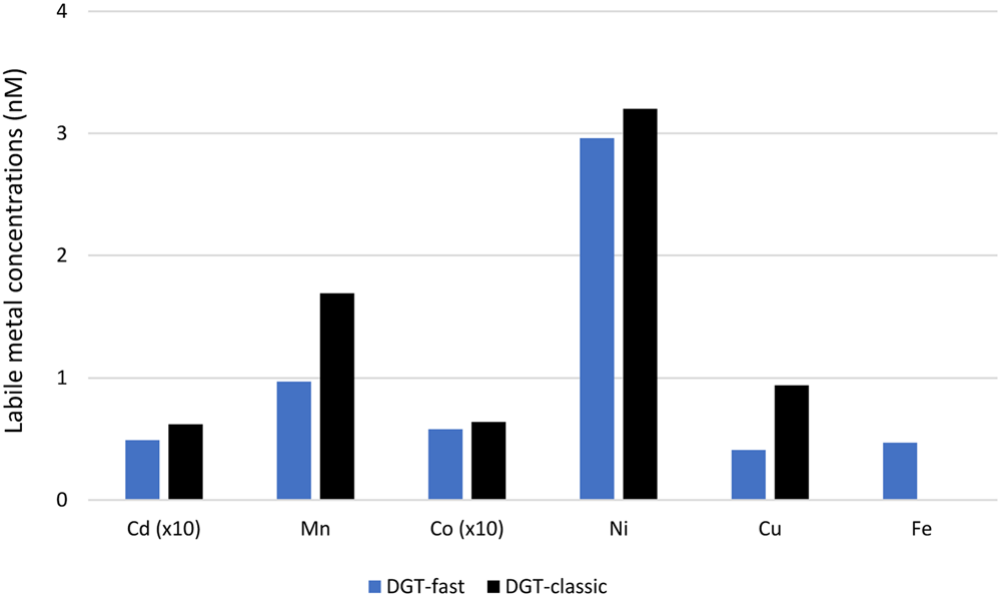
Labile metal concentrations obtained by fast and classic DGTs in the Mediterranean Sea.

If there was auto-contamination from the glider, the DGTs mounted at the back of the glider would accumulate higher amounts of trace metals than those mounted at the front. Figure 2 and Table S1 show the results expressed as *c*_DGT_ calculated from the accumulated mass *M*, of each metal as

1 $${c}_{{\rm{DGT}}}=\frac{M\,{\delta }^{{\rm{g}}}}{{D}_{{\rm{M}}}\,t\,A}$$

where *M* (nmoles) is the accumulated mass of analyte, *D*_M_ (cm^2^ s^−1^) the diffusion coefficient, *t* (s) the deployment time, *A* (cm^2^) the effective area and *δ*^g^ (cm) the thickness of diffusive domain for each type of sampler.

A statistical test (paired t-test at *p* = 0.05) indicates that there is no difference between the results obtained with DGTs at the head and at the tail of the glider, except for Mn and Cu, where the values at the glider’s head are somewhat higher than at its tail, excluding auto-contamination. Examination of the sample to blank signal ratios indicates that, for the selected metals, the sensitivity achieved during the campaign is good to very good, with ratios between 4 and 38. The major contribution to those blanks comes from background levels in the resin gel. This performance is impressive given that there was no pretreatment of the resin.

The only published use of DGT samplers in the open ocean, rather than coastal systems, estuaries and fjords, is a previous study in the Southern Ocean where we compared total dissolved with labile trace metal concentrations obtained by DGT^21^. The percentages of labile (*c*_DGT_) to total dissolved metal concentration were lowest for Fe and Cu, showing that these metals are predominantly present in the form of colloids and/or strong metal-ligand complexes, which are generally less bio-available to phytoplankton. For example, weakly complexed ferric hydrolysis species Fe(III) are reduced at orders of magnitude higher rates than strongly bound Fe-siderophore chelates and are, thus, much more accessible for cellular uptake^12,28,29^. But, even for Cd, 40% of the dissolved load was in a non-labile form in the Southern Ocean. The ratio of highly labile metal concentrations $({c}_{{\rm{DGT}}}^{{\rm{fast}}})$ that we observed in the Mediterranean Sea to total dissolved trace metal concentrations observed by Yoon *et al*.^18^ in a similar area is lower than 50% for Cu and Fe, 65% for Cd and 90% for Ni. We can only compare our labile Fe result (${c}_{{\rm{DGT}}}^{{\rm{fast}}}$ = 0.47 nM) with dissolved Fe results reported by Guieu *et al*.^23^ which are in the range 0.26–2.72 nM for a similar area in the Mediterranean Sea, but were obtained with a hydroxy-quinoline column for pre-concentration and chemiluminescence detection. During several campaigns in the Southern Ocean (Table 1) we also found a good agreement between our Fe-DGT results obtained via shipboard incubations in large sea water volumes with those obtained using flow injection with chemiluminescence detection^15,19^, but neither measurement was *in situ* and there was no discussion of the lability degree of the measured species.

**Table 1:** Comparison of our results with literature data for the Mediterranean Sea and Southern Ocean. Concentrations in nM. Labile* and total dissolved concentration**.

	Cd	Mn	Fe	Co	Ni	Cu
Our results-Fast DGT* Western Medit. Sea	0.049	0.97	0.47	0.058	2.95	0.41
Our results-Classic DGT* Western Medit. Sea	0.062	1.69		0.064	3.2	0.94
Guieu *et al*. 2002** Western Medit. Sea			0.27–1.99			
Yoon *et al*. 1999** Western Medit. Sea	0.075		1.4		3.3	1.7
Ebling and Landing 2015** Western Medit. Sea	0.065	6.42	5.54		4.9	4.53
Dulaquais *et al*. 2017** Western Medit. Sea				0.120		
Baeyens *et al*. 2011* Southern Ocean	0.02–0.15	0.08–0.28	0.1–0.7	0.002–0.014	0.4–2.3	0.1–0.36
Butler *et al*. 2013** Southern Ocean	0.005–0.27		0.2–0.5	0.01–0.03	2.5–6.2	0.25–1.00
Stockdale *et al*. 2016 free metal	4%	45%	5.3 10^−13^	16%	10%	0.1%
Stockdale *et al*. 2011 and 2016 inorganic complex	96.0%	54.4%	1.20%	84%	89.2%	2%
Stockdale *et al*. 2011 and 2016 organic complex	0.05%	0.6%	98.8%	0.021%	0.80%	98%

## Discussion

### Potential growth limitation

**Figure 3:**
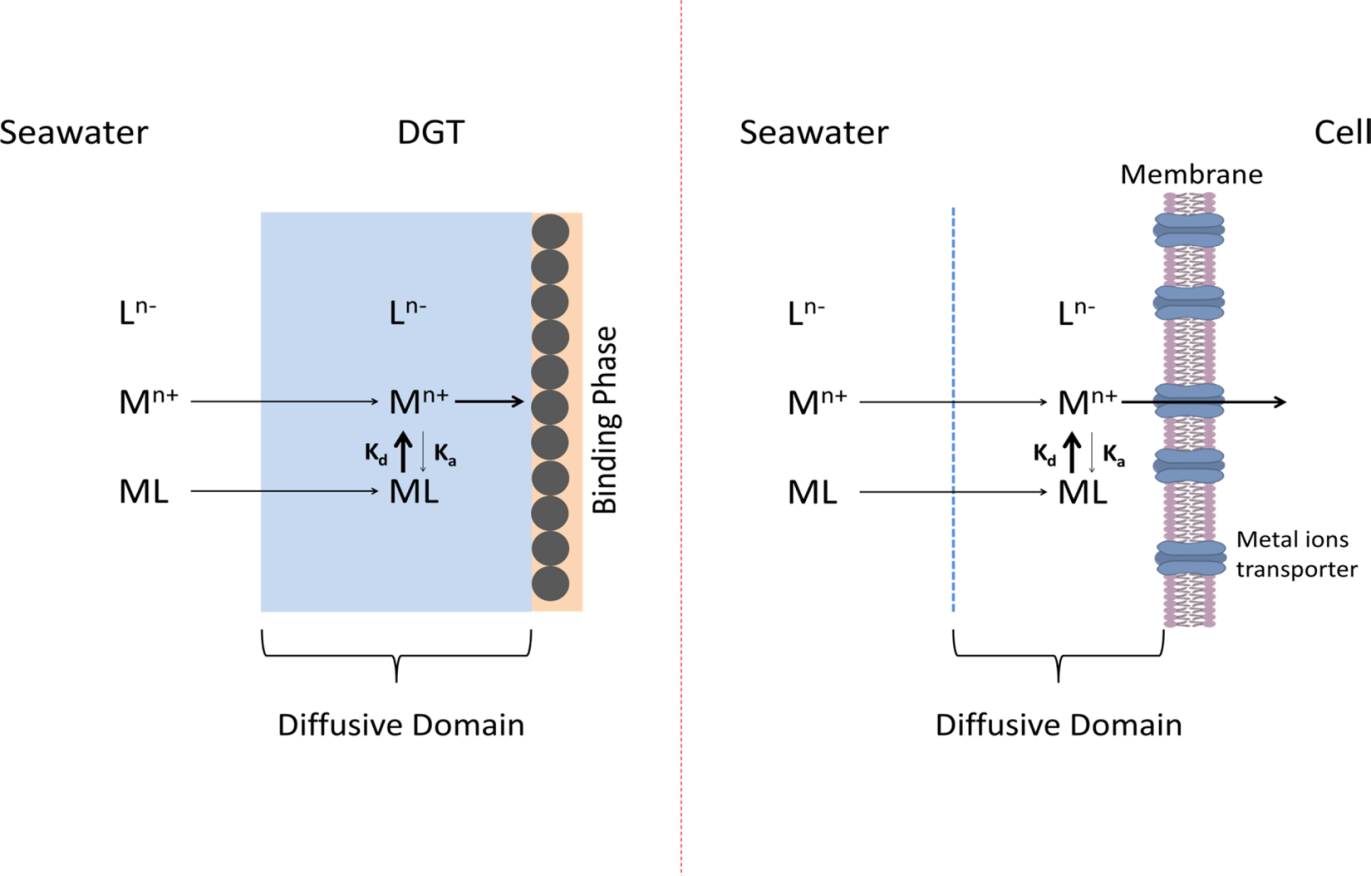
Dissociation of labile metal complexes and internalization of free ions by a plankton cell. Comparison with a DGT sampler.

Plankton growth in the Mediterranean Sea is usually limited due to a lack of phosphate^30^, but trace metals were not considered in that study. Trace metal nutrients, like major nutrients, are taken up intracellularly by specialized transport proteins on the cytoplasmic membrane of algal cells. Virtually all of these proteins act as pumps and require energy for intracellular transport. With some exceptions, the binding of metals to the receptor sites on these proteins is determined by the concentration of free aquated metal ions or when the internalization step is faster than the transport of free metal ions, the binding to the receptor is influenced by the free metal released from the complexes which effectively buffer the metal consumption by the receptors. Thus, as a general case, the internalization is dependent on the labile metal concentration, something estimated by *c*_DGT_, used in our work (Fig. 3). In many cases, it has been shown that metal inorganic complexes with Cl^−^, OH^−^, and CO_2_^−3^ are labile^31,32^. Usually, chelation by organic ligands renders complexes with higher stability. According to Eigen mechanism, these complexes tend to be less labile due to their slow dissociation kinetics. Thus, complexes with organic ligands generally decrease metal uptake and chemical speciation is extremely important in regulating the cellular uptake of metals^33^. As an example, we mention the iron uptake process. There is mounting evidence for the utilization of a high-affinity transport system that accesses a variety of Fe(III) coordination species (including Fe(III) and ferric chelates) via reduction to Fe(II)^34–36^. The released Fe(II) binds to Fe(II) receptors on specific transmembrane proteins, which transport the iron into the cell. This intracellular transport involves the reoxidation of bound Fe(II) to Fe(III) by a copper protein (a multi-Cu oxidase^37^). The ability of this transport system to access iron is dependent on the ease of reduction of ferric complex species, which is inversely related to the stability of the Fe(III) coordination complex^34^. There are some experimental data that support the direct internalization of organic complexes without dissociation, but very few cases have been reported. An example is the direct incorporation of iron complexes with siderophores via Ton B dependent receptors^13,14^, but this process uses cellular energy to drive active transport at the outer membrane and the question arises if this process can compete with the internalization of free metal ions. Weakly complexed ferric hydrolysis species Fe(III) are reduced at orders of magnitude higher rates than strongly bound Fe-siderophore chelates and are, thus, much more accessible for cellular uptake^12,28,29^.

It is thus very important to assess kinetically labile dissolved metal species because they produce free aquated metal ions in the diffusive domain around the plankton cell (Fig. 3). By using DGTs of various diffusive domain thicknesses, we assessed concentrations of very labile metal complexes (Table S1 and Fig. 2) that were also used in the estimation of potential limiting micronutrients (dissociation time about 3 minutes) and moderate labile metal complexes (dissociation time about 40 minutes). Both DGTs and phytoplankton cells have a diffusive domain which allows dissociation of metal complexes prior to their uptake, as facilitated by their residence time in that diffusive domain (see Fig. S2 for Fe, Co and Mn). Cellular metal quotas for diatoms, autotrophic flagellates and autotrophic picoplankton were retrieved from the study of Twining *et al*.^38^ in the North Atlantic Ocean. They can be used for our calculations of limitations in the Mediterranean Sea because there are arguments for the Mediterranean Sea values being even higher. Variability in ocean geochemistry has not only resulted in stoichiometric differences (cellular quotas) among taxa^39^, but the same taxa, in this case diatoms, living in different oceanic basins, also show stoichiometric differences (Fig. S2). The higher the metal concentrations in the seawater, the higher their cellular quotas: both are lowest in the Southern Ocean and highest in the North Atlantic, which receives higher inputs from rivers and atmospheric deposition than the other oceans^40^. In the Mediterranean Sea, the metal concentrations in the seawater are still higher than in the North Atlantic Ocean, which would be expected to produce even higher cellular quotas in Mediterranean Sea diatoms.

Once cellular quotas in phytoplankton and micronutrient concentrations in seawater are known, the potentially limiting elements can be determined. When cellular stoichiometry and micronutrient concentrations are sufficiently stable over the study period, the micronutrient with the smallest pool in solution versus its cellular quota should limit the rate of new biomass production (see for example Moore *et al*.^39^). Phosphorous is generally considered as the growth limiting element in the Mediterranean Sea (0.04 µM^30^), but to our knowledge no trace metal nutrients were compared. It is thus possible to normalize seawater and intracellular micronutrient concentrations to P to determine if some micronutrients are more limiting or co-limiting. In diatoms the ratios of Fe/P, Co/P and Cu/P are respectively 76, 3.1 and 10 (all in nmol/µmol) while these ratios in seawater are respectively 12, 1.5 and 10 (all in nmol/µmol). With seawater Me/P ratios that are respectively 6 times lower (Fe/P), 2 times lower (Co/P) and the same (Cu/P) than their ratios in diatoms (cellular quotas in the Mediterranean Sea may be even higher than in the North Atlantic Ocean as explained before), Fe, Co and Cu are potentially more limiting or co-limiting micronutrients than P. The cellular quotas for diatoms in the North Atlantic are obtained over a period of 58 days and are fairly stable with a variability of 20% for Fe/P, 39% for Co/P and 60% for Cu/P. Using cellular quotas that are 20% lower for Fe and 39% for Co, does not change the potential limiting effect of both elements for diatoms in the Mediterranean Sea. For all other plankton taxa and for Cd, Mn and Ni in diatoms, the cellular quotas are lower than their ratios in seawater. Thus, for flagellates and picoplankton, phosphate remains the main growth limiting element. Is the lability of the trace metals measured by the DGTs, however, as high as that of phosphate, which is present as free ion and, thus, directly available to plankton? Therefore, a kinetic analysis of the metal complex pools in the fast and in the classic DGT devices, including their dissociation rates and half-lives, was made.

### Average lability and dissociation rate of inorganic and organic metal complexes

Using literature data (such as total dissolved concentration of each metal, *c*_T,M_, and its percentages free, inorganic and organic), we can apportion, as a first approximation, the total dissolved amount into three pools: the free metal pool ${c}_{{\rm{M}}}^{\ast }$, an inorganic pool ${c}_{{{\rm{ML}}}_{{\rm{in}}}}^{\ast }$ and an organic pool ${c}_{{{\rm{ML}}}_{{\rm{org}}}}^{\ast }$.

2 $${c}_{{\rm{T}},{\rm{M}}}={c}_{{\rm{M}}}^{\ast }+{c}_{{{\rm{ML}}}_{{\rm{in}}}}^{\ast }+{c}_{{{\rm{ML}}}_{{\rm{org}}}}^{\ast }$$

The superscript * indicates bulk values, while L_in_ and L_org_ are the ligands associated to each pool. The lability degrees and dissociation rates of the metal complexes that we calculate here below are, thus, averages for the complexes existing in each of these 2 pools.

It has been shown^17,41,42^ that the DGT concentration is a summation of the labile fractions, each weighted by a normalized diffusion coefficient ε_*j*_ (the ratio between the diffusion coefficient of the pool over the diffusion coefficient of the metal). For the fast and classic DGTs we can write:

3 $${c}_{{\rm{DGT}}}^{{\rm{classic}}}={c}_{{\rm{M}}}^{\ast }+{\varepsilon }_{{\rm{in}}}{\xi }_{{\rm{in}}}^{c}{c}_{{{\rm{ML}}}_{{\rm{in}}}}^{\ast }+{\varepsilon }_{{\rm{org}}}{\xi }_{{\rm{org}}}^{c}{c}_{{{\rm{ML}}}_{{\rm{org}}}}^{\ast }$$

4 $${c}_{{\rm{DGT}}}^{{\rm{fast}}}={c}_{{\rm{M}}}^{\ast }+{\varepsilon }_{{\rm{in}}}{\xi }_{{\rm{in}}}^{{\rm{f}}}{c}_{{{\rm{ML}}}_{{\rm{in}}}}^{\ast }+{\varepsilon }_{{\rm{org}}}{\xi }_{{\rm{org}}}^{{\rm{f}}}{c}_{{{\rm{ML}}}_{{\rm{org}}}}^{\ast }$$

Here *ξ*_*j*_ is the lability degree (between 0, for inert complexes, and 1, for fully labile complexes) for each metal complex pool (*j* = in or org) in the fast or classic DGT (superscripts f or c). The lability degree quantifies the contribution of a given complex (or ensemble of complexes) to the flux received by a microorganism or a sensor in comparison with the maximum possible contribution of this complex if it was fully labile^43^.

${c}_{{\rm{DGT}}}^{{\rm{classic}}}$ and ${c}_{{\rm{DGT}}}^{{\rm{fast}}}$ were measured *in situ* with the DGT devices mounted on the SeaExplorer. Total dissolved Cd, Cu, Fe and Mn concentrations (*c*_T,M_) have been recently measured by Ebling and Landing^44^ in an area close to our sampling site. Total dissolved Co was measured by Dulaquais *et al*.^45^ in the whole Basin of the Mediterranean Sea. We used their results for the central-western Basin while the percentages of free metal, inorganic complexes (identified with pool in) and organic complexes (pool org) were taken from Stockdale *et al*.^46,47^. Calculated values of the labilities and dissociation rates of the metal complex pools are summarized in Table 2, while details of the calculations can be found in METHODS and MATERIALS.

**Table 2:** Calculated labilities (*ξ*) and dissociation rates (*k*_d_) of metal complexes in the Mediterranean Sea.

Element	*c*_T,M_	Free metal	DGT-fast	DGT-classic	ε_1_	${{\boldsymbol{\xi }}}_{{\bf{1}}}^{{\boldsymbol{c}}}$	${{\boldsymbol{\xi }}}_{{\bf{1}}}^{{\bf{f}}}$	*k*_d,1_	ε_2_	${{\boldsymbol{\xi }}}_{{\bf{2}}}^{{\boldsymbol{c}}}$	${{\boldsymbol{\xi }}}_{{\bf{2}}}^{{\bf{f}}}$	*k*_d,2_
	(nM)	(nM)	(nM)	(nM)				s^−1^				s^−1^
Cd	0.066	0.0029	0.049	0.062	1.07	0.89	0.694	0.019	—	—	—	—
Fe	5.54	5.3 × 10^−13^	0.47	—	—	—	—	—	0.57	—	0.152	0.00043
Ni	4.9	0.49	2.95	3.18	0.64	0.96	0.88	0.089	—	—	—	—
Co	0.12	0.019	0.058	0.064	0.48	0.94	0.81	0.019	—	—	—	—
Cu	4.53	0.0044	0.41	0.98	1.0	0.89	0.70	0.018	0.57	0.36	0.14	0.00035

*c*_T,M_ is the total dissolved metal concentration (Co from Dulaquais *et al*. (2017 and the other metals from Ebling and Landing (2015), free metal concentrations are from Stockdale *et al*. (2011; 2016) and ε is the ratio of the diffusion coefficient of the metal complex to that of the metal ion.

According to Stockdale *et al*.^47^, Cd speciation in the ocean is dominated by inorganic, chloride complexes (more than 93%). Even in the Scheldt estuary, which was historically heavily polluted by metals and organic matter^48^, inorganic Cd-chloride complexes were largely dominant in the downstream estuary. At low levels in open ocean, Cd has been shown to be significantly complexed by strong ligands^49^. If this were the case, there would be no significant contribution from free metal and the inorganic pool and hence a large difference between total dissolved Cd and DGT-Cd. This was not observed in our study of the Mediterranean Sea where metal concentrations are generally higher than in the central North Pacific Ocean. Additionally, if the mobility of organic complexes is smaller than that of the inorganic pool, their contribution to the availability will be even more reduced. Thus, we will assume that the contribution of CdL_org_ to *c*_DGT_ is negligible. So,

5 $${c}_{{\rm{DGT}},{\rm{Cd}}}^{{\rm{classic}}}={c}_{{\rm{Cd}}}^{\ast }+{\varepsilon }_{{\rm{in}},{\rm{Cd}}}{\xi }_{{\rm{in}},{\rm{Cd}}}^{c}{c}_{{{\rm{CdL}}}_{{\rm{in}}}}^{\ast }+{\varepsilon }_{{\rm{org}},{\rm{Cd}}}{\xi }_{{\rm{org}},{\rm{Cd}}}^{c}{c}_{{{\rm{CdL}}}_{{\rm{org}}}}^{\ast }\approx {c}_{{\rm{Cd}}}^{\ast }+{\varepsilon }_{{\rm{in}},{\rm{Cd}}}{\xi }_{{\rm{in}},{\rm{Cd}}}^{c}{c}_{{{\rm{CdL}}}_{{\rm{in}}}}^{\ast }$$

6 $${c}_{{\rm{DGT}},{\rm{Cd}}}^{{\rm{fast}}}\approx {c}_{{\rm{Cd}}}^{\ast }+{\varepsilon }_{{\rm{in}},{\rm{Cd}}}{\xi }_{{\rm{in}},{\rm{Cd}}}^{{\rm{f}}}{c}_{{{\rm{CdL}}}_{{\rm{in}}}}^{\ast }$$

7 $${\xi }_{{\rm{in}},{\rm{Cd}}}^{c}=f({k}_{{\rm{d}},{\rm{in}},{\rm{Cd}}},{\delta }^{g,c})$$

8 $${\xi }_{{\rm{in}},{\rm{Cd}}}^{{\rm{f}}}=f({k}_{{\rm{d}},{\rm{in}},{\rm{Cd}}},{\delta }^{{\rm{g}},{\rm{f}}})$$

Equations (7) and (8) relate the lability degree of pool in, in each DGT with the pertinent thickness of the diffusive domain and complex dissociation rate. See equation (12) further in METHODS and MATERIALS for the calculation indicated by the “f” function. Four equations (5) to (8), with 4 unknowns: ${\varepsilon }_{{\rm{in}},{\rm{Cd}}}$, ${k}_{{\rm{d}},{\rm{in}},{\rm{Cd}}}$, ${\xi }_{{\rm{in}},{\rm{Cd}}}^{{\rm{c}}}$ and ${\xi }_{{\rm{in}},{\rm{Cd}}}^{{\rm{f}}}$, have to be solved simultaneously. Using *c*_T,Cd_ = 0.0655 nM^44^ and 4% free, 96% complexed^47^, the solution yields the following results: ${\varepsilon }_{{\rm{in}},{\rm{Cd}}}$ = 1.07, ${\xi }_{{\rm{in}},{\rm{Cd}}}^{{\rm{c}}}$ = 0.89, ${\xi }_{{\rm{in}},{\rm{Cd}}}^{{\rm{f}}}$ = 0.69, ${k}_{{\rm{d}},{\rm{in}},{\rm{Cd}}}$ = 0.019 s^−1^. These values are quite reasonable. For instance, it is well-known that some halide complexes of Cd diffuse faster than the free ion (due to a the replacement of a water hydration molecule by the smaller halide), see Serrano^50^ and references therein, so the fact that ${\varepsilon }_{{\rm{in}},{\rm{Cd}}}$ > 1 is plausible. Considering possible inaccuracies in the input values, ${\xi }_{{\rm{in}},{\rm{Cd}}}^{{\rm{f}}}$ is reassuringly close to the unity expected for full lability of inorganic Cd-complexes.

Cu is not only a micronutrient for phytoplankton, as the free ion is toxic at even very low concentrations. For *T.pseudonana*, copper was inhibitory at pCu values below 10.7^51^. The chemical speciation of Cu in seawater is dominated by organic complexation (e. g.^52^) leading to a very low free Cu concentration. According to Stockdale *et al*.^47^, the proportion of inorganic complexes is about 2% against 98% of organic complexes. The identity of the organic ligands is still poorly known^21^, but Cu-binding thiols have been identified, emanating from reducing marine sediments^53^ or produced and exuded by phytoplankton^54^. Humic acids are known to bind Cu in both freshwater and seawater (e.g.^21^) and may represent a component of the organic pool of copper-binding ligands. The two pools used in this work are to be distinguished from the so-called L_1_ and L_2_ ligands often invoked to model “organically dominated” speciation in natural waters. Town and Filella^55^ questioned the real existence of just 2 distinct groups of ligands. They made a critical analysis and interpretation of data from 77 studies between 1975 and 1998 of complexation (stability constants and complexation capacities) of copper in seawater and freshwater samples. Stronger binding sites are utilized at lower Cu concentrations and progressively weaker sites contribute to complexation at higher metal concentrations. Stability constants give us an idea about the strength of a metal complex, but not about the rate it will dissociate and be available to organisms, so, kinetic information, as sought in this work, is helpful for a deeper understanding of bioavailability. For this case of Cu, both pools have to be considered as contributing to *c*_DGT_ and accordingly, we should solve a system of 6 equations, including equations (3), (4), (7), (8) (re-written replacing Cd with Cu) and equations (9) and (10) here below:

9 $${\xi }_{{\rm{org}},{\rm{Cu}}}^{{\rm{c}}}=f({k}_{{\rm{d}},{\rm{org}},{\rm{Cu}}},{\delta }^{{\rm{g}},{\rm{c}}})$$

10 $${\xi }_{{\rm{org}},{\rm{Cu}}}^{{\rm{f}}}=f({k}_{{\rm{d}},{\rm{org}},{\rm{Cu}}},{\delta }^{{\rm{g}},{\rm{f}}})$$

8 unknowns are involved in these equations: ${\varepsilon }_{{\rm{in}},{\rm{Cu}}}$, ${k}_{{\rm{d}},{\rm{in}},{\rm{Cu}}}$, ${\varepsilon }_{{\rm{org}},{\rm{Cu}}}$, ${k}_{{\rm{d}},{\rm{org}},{\rm{Cu}}}$,${\xi }_{{\rm{in}},{\rm{Cu}}}^{{\rm{c}}}$, ${\xi }_{{\rm{in}},{\rm{Cu}}}^{{\rm{f}}}$, ${\xi }_{{\rm{org}},{\rm{Cu}}}^{{\rm{c}}}$ and ${\xi }_{{\rm{org}},{\rm{Cu}}}^{{\rm{f}}}$. However, the inorganic Cu complexes (pool in) are expected to have a similar ${\varepsilon }_{{\rm{in}},{\rm{Cu}}}$ value as the inorganic Cd complexes, allowing us to assume that ${\varepsilon }_{{\rm{in}},{\rm{Cu}}}$ = 1. As diffusion coefficient for pool 2 we take the minimum value, 3.48 10^−10^ m^2^s^−1^ (which lies in the middle of previously reported values for humic acids), given by Balch and Gueguen^56^ for humic acid at 25 °C and divide it by the free metal diffusion coefficient at the same temperature. This yields a value for ${\varepsilon }_{{\rm{org}},{\rm{Cu}}}$ around 0.57. We can then calculate the 6 remaining parameters with the 6 equations above (3, 4 and 7–10).

The retrieved solution is: ${\xi }_{{\rm{in}},{\rm{Cu}}}^{{\rm{c}}}$ = 0.89, ${\xi }_{{\rm{in}},{\rm{Cu}}}^{{\rm{f}}}$ = 0.70, ${k}_{{\rm{d}},{\rm{in}},{\rm{Cu}}}$ = 0.018 s^−1^, ${\xi }_{{\rm{org}},{\rm{Cu}}}^{{\rm{c}}}$ = 0.36, ${\xi }_{{\rm{org}},{\rm{Cu}}}^{{\rm{f}}}$ = 0.14 and ${k}_{{\rm{d}},{\rm{org}},{\rm{Cu}}}$_ = _0.00035 s^−1^. The half-life of the inorganic Cu pool is 36 s (1.7% of the total dissolved Cu burden of 4.5 nM is inorganic; this corresponds to 0.08 nM or 22% of the fast DGT concentration), confirming that this pool is almost fully labile. The organic pool, which constitutes 98% of the total dissolved Cu burden has a half-life of 33 min.

These solutions look quite acceptable because: for both pools, the classic labilities are higher than the fast ones (${\xi }_{{\rm{in}}}^{{\rm{c}}} > {\xi }_{{\rm{in}}}^{{\rm{f}}}$ and ${\xi }_{{\rm{org}}}^{{\rm{c}}} > {\xi }_{{\rm{org}}}^{{\rm{f}}}$), the labilities of pool org are for both DGT devices lower than those of pool in (${\xi }_{{\rm{org}}}^{{\rm{c}}} > {\xi }_{{\rm{in}}}^{{\rm{c}}}$ and ${\xi }_{{\rm{org}}}^{{\rm{f}}} > {\xi }_{{\rm{in}}}^{{\rm{f}}}$) and the dissociation rate of the copper complexes in pool in, is higher than those of pool org (${k}_{{\rm{d}},{\rm{in}}} > {k}_{{\rm{d}},{\rm{org}}}$).

The solubility of iron in seawater is very low, therefore much of the dissolved iron is present as small colloids (>0.02 μm) and, thus, likely subject to aggregation and scavenging removal. No data could be obtained for Fe with the classic DGT, so no “general” treatment can be applied. However, according to Stockdale *et al*.^47^, soluble Fe-complexes are 98.8% organic, so we can neglect other contributions and write:

11 $${c}_{{\rm{DGT}},{\rm{Fe}}}^{{\rm{fast}}}\approx {\xi }_{{\rm{org}},{\rm{Fe}}}{\varepsilon }_{{\rm{org}},{\rm{Fe}}}\,{c}_{{{\rm{FeL}}}_{{\rm{org}}}}^{\ast }$$

Taking *c*_T,Fe_ = 5.54 nM^41^ and ${\varepsilon }_{{\rm{org}},{\rm{Fe}}}$ = 0.57 (as in the case of Cu), one gets ${\xi }_{{\rm{org}},{\rm{Fe}}}^{{\rm{f}}}$ = 0.152. More robust is that we find that the product ${\varepsilon }_{{\rm{org}},{\rm{Fe}}}{\xi }_{{\rm{org}},{\rm{Fe}}}^{{\rm{f}}}$ is 0.0087, reflecting the low mobility and lability of this pool. Using a *D*_Fe_ of 5.02 × 10^−10^ m^2^s^−1^ (source is DGT research website) we calculated a ${k}_{{\rm{d}},{\rm{org}},{\rm{Fe}}}$ of 0.00043 s^−1^. In the Celtic Sea, Abualhaija and van den Berg^57^ found a dissociation rate of the complex of iron with the natural ligand of 0.00133 s^−1^, which is of the same order of magnitude as our estimate in the Mediterranean Sea. The half-life of the Fe organic complex pool is 27 min.

Dissolved Co has the smallest organic pool of all trace metals here considered (0.02%), so the same mathematical treatment as for Cd can be applied. The here calculated ${\varepsilon }_{{\rm{in}},{\rm{Co}}}$ value appears relatively low (0.48), but the chemistry of cobalt is highly complex. Cobalt exists in seawater as soluble Co(II) or as Co(III), which forms insoluble oxides at the pH of seawater^12^ or in some cases dissolved cobalt can be extensively complexed by strong organic ligands in seawater^58,59^. The calculated lability and dissociation rate parameters of ${\xi }_{{\rm{in}},{\rm{Co}}}^{{\rm{c}}}$ = 0.94, ${\xi }_{{\rm{in}},{\rm{Co}}}^{{\rm{f}}}$ = 0.81 and ${k}_{{\rm{d}},{\rm{in}},{\rm{Co}}}$ = 0.019 s^−1^ are consistent with Co being almost fully labile and with a similar half-life of the complexes as the inorganic Cu pool.

Dissolved Ni is 99% in the inorganic pool, as free ions or as inorganic complexes^44^. Ni data can consequently be treated in the same way as dissolved Cd and Co. The following results were obtained: ${\varepsilon }_{{\rm{in}},{\rm{Ni}}}$ = 0.64 (indicating that in this almost fully labile pool, there are also complexes with smaller diffusion coefficient than the free metal), ${\xi }_{{\rm{in}},{\rm{Ni}}}^{{\rm{c}}}$ = 0.96, ${\xi }_{{\rm{in}},{\rm{Ni}}}^{{\rm{f}}}$ = 0.88 and ${k}_{{\rm{d}},{\rm{in}},{\rm{Ni}}}$ = 0.089 s^−1^.

Manganese, which is a redox sensitive element, shows a quite different behavior in aquatic environments than most other trace metals. Solid MnO_2_ is the stable form of manganese in oxygenated waters, while dissolved Mn is thought to be dominated by metastable Mn(II and III). Moreover, of all elements that we determined, it has the lowest affinity for the iminodiacetic groups of the Chelex resin. This makes its measurement by DGT the most susceptible to competition and saturation effects at the salinity of seawater, which could possibly explain the difference between the fast and classic DGT results^60^. In the last decade, soluble Mn has been re-evaluated to include soluble Mn (III)-complexes^61^ and recently Oldham *et al*.^25^ found that humic ligands are responsible for up to 100% of the Mn(III)-complexes and up to 86% of total dissolved manganese in oxygenated marine waters. A possible explanation for our data is that these Mn(III) species are partially labile, which provides additional insight into this intriguing Mn chemistry. However, as this role of organic Mn complexes in marine waters is speculative, we could not justify retrieving a solution for the lability and dissociation rate of the Mn complexes pool.

## Conclusions

This work has demonstrated that it is possible to determine ultra-low labile concentrations of trace metals Cd, Co, Cu, Fe, Mn and Ni in the Mediterranean Sea using a SeaExplorer glider that has been modified for the inclusion of DGT samplers. The *in situ* differentiation of metal complexes with different labilities is a very valuable advantage of the glider-DGT combination, overcoming problems associated with changes of the metal complexes’ composition during treatment or storage^24,26^. It will be advantageous in future to deploy DGTs with a wider range of diffusive and resin layer thicknesses^41,42^ because this will allow us to make a better distinction between the various pools of metal complexes existing in natural marine waters.

Concentrations of many nutrients and micronutrients in the oceans are potentially close to co-limiting^11,62^, but our predictive ability is largely clouded by the large uncertainties surrounding the diversity of the chemical species of each nutrient, and the diversity of biological strategies phytoplankton use to acquire those various chemical forms^11^. Since the diffusion domain thickness is a critical parameter in setting lability ranges and DGT uniquely allows easy variation of this parameter, a detailed speciation of highly labile and moderately labile complexes can be performed *in situ*. In the Mediterranean Sea, inorganic complexes of Cd, Co, Ni and Cu appeared to be highly labile. Between 70 and 88% of these inorganic complexes were dissociated in the fast DGT while this percentage increased to more than 89% in the classic DGT. The organic pools of Fe (99%) and Cu (98%) show a very different behavior with respectively only 15 and 14% dissociated in the fast DGT and with half-lives of 27 and 33 min. On the basis of their labile metal concentrations measured by the fast DGT, we discovered that Fe, Co and even Cu can be potentially limiting or co-limiting to diatom growth. For flagellates and picoplankton, phosphorus remains the main growth limiting element. We also observed that Fe and Cu complexes, which are mainly formed by organic ligands (>98%), dissociate slower than Co, Cd and Ni complexes, which mainly consist of inorganic ligands, while the free ion fraction of those metals is also much higher than that of Fe and Cu (free ions and inorganic complexes represent more than 99% of the dissolved concentrations of Co, Cd and Ni). This supports the potential growth-limiting effect of Fe and Cu versus phosphorus (phosphate), because the latter element is present as a free ion and, thus, directly available for plankton.

## Methods and Materials

### Modified seaexplorer

The SeaExplorer (SEA010 from ALSEAMAR, France) is a type of autonomous underwater vehicle that uses small changes in its buoyancy in conjunction with wings to convert vertical motion to horizontal, and thereby propels itself forward with very low power consumption. This glider can perform sampling missions that last from weeks to months and cover thousands of kilometers. However, the commercially available instrument is not designed for the determination of trace metals in the ocean. Therefore, we equipped its nose (Fig. 1) and its tail with Diffusive Gradients in Thin Films (DGTs), and arranged that the nose was completely empty inside. The SeaExplorer is constructed in aluminum (the middle section is coated with a polymer) and anodized aluminum, the nose and tail in polyurethane. The concentrations of impurities in Al and anodized Al materials, used for the construction of the SeaExplorer, are shown in Table S2. The composition of the Al and anodized Al is different, with highest trace metal contents for the anodized plate. Leaching of trace metals from the anodized SeaExplorer’s sealing plates in Milli-Q water during several days was indistinguishable from the Milli-Q blank. The impurities in the polyurethane material are lower than in the Al materials (Table S2). The DGT probes are inserted in the polyurethane material, which therefore constitutes a lower risk for contamination. Several leaching solutions (Milli-Q water and acidic solutions) were tested: the highest amounts of trace metals were found when using strong acids such as HCl or HNO_3_, but in Milli-Q water no leaching was observed. However, as an additional measure all metal surfaces including the anodized Al plates were coated with a metal free resin (Armourgard ST anti-corrosion steel primer and syntac Epoxy Resin from Reactive Resins Ltd, Cornwall, UK).

### Diffusive gradients in thin films (DGTs)

#### Gel preparations

A polyacrylamide hydrogel consisting of 15% acrylamide (Merck, Belgium) and 0.3% agarose derived cross linker (DGT Research Ltd., UK) was used as a diffusive hydrogel in a DGT probe. The recipe for making the diffusive gel can be found elsewhere^63^. Chelex-100 was used to prepare the resin gels according to the recipe obtained from DGT research Ltd: 1.6 gram chelex-100 resin was added to 4 mL of gel solution (mixed in advance and stored in a refrigerator at 4 °C), which is a mixture of acrylamide (40%, Merck, Belgium), acrylamide cross-linker (2%, DGT Research Ltd, UK) and MilliQ water. 24 µL ammonium persulfate (1%, Merck, Belgium) and 6 µL N,N,N,N-tetraethylenediamine (TEMED, Merck, Belgium) were added to the mixture and the mixed solution was casted into a glass assembly separated with a fixed thickness of Teflon spacer (0.04 cm). The assembly was placed in an oven at 45 °C for 1 h, and then the gel was peeled from the glass plates and hydrated in MilliQ water for at least one day before use.

#### Assembling the DGT samplers

Diffusive and resin gels were cut into 2.5 cm diameter discs with a plexi-glass gel cutter and the resin gel was first mounted on the piston base. The diffusive gel was, then, placed on top of the resin gel and covered by a 0.45 µm Millipore Durapore membrane filter (HVLP, Millipore, 0.0125 cm thickness). For the fast DGTs, the resin gel was placed on top of the diffusive gel and directly covered by the Millipore filter, so that the diffusion domain was just the filter thickness plus the diffusive boundary layer. The cap was then placed on the piston and pressed down to the bottom of the base to seal the probe. Both, in fast and classic DGT, the effective exposure area to the sea water (*A*) is 3.14 cm^2^.

### Analysis and calculation of labile trace metals

The metal amounts captured by the chelex resin gel were eluted with 1 mL of 1 M nitric acid and further 10 times diluted (0.5 mL extract in 5 mL of Milli-Q). Elution factors from Chelex-100 are 0.8^63,64^. This solution was then analyzed with High Resolution ICP-MS (Thermo Finnigan Element II) and compared to calibration curves of the trace metals made from appropriate dilutions of an acidified multi-element stock solution (Merck, ICPMS standard XIII). Knowing the metal amount (*M*) accumulated in the resin, the DGT concentrations in the seawater can be calculated invoking Fick’s first diffusion law^63,64^, which under steady state and perfect sink conditions leads to equation (1). One of the variables in equation (1) is the diffusive domain thickness (*δ*^g,c^, and *δ*^g,f^). The thickness of the hydrogel and the filter are known, but that of the diffusive boundary layer (DBL) has to be estimated. Recently we measured the DBL in a well-mixed water-effluent^65^. Since the glider is moving all the time and the surface water is well-mixed, a similar DBL of 0.02 cm was adopted. The variables in equation (1) have the following values: the diffusive domain of the fast DGT is 0.0325 cm (0.0125 cm filter plus 0.02 cm DBL) and of the classic DGT is 0.1125 cm (0.08 cm hydrogel plus the filter and DBL thicknesses); the exposure time is 576 h and the diffusion coefficients in (cm^2^ s^−1^) at 18 °C used (source is DGT-research website): Cd (5.00 × 10^−6^); Mn (4.80 × 10^−6^); Fe (5.02 × 10^−6^); Co (4.88 × 10^−6^); Ni (4.74 × 10^−6^); Cu (5.12 × 10^−6^).

### Field cruise

A 24 days survey (mission start time: 07/06/2016 09:09 and mission end time: 01/07/2016 10:08) of the SeaExplorer glider (Fig. 2) has been performed in the surface layer (from surface to 100 m of depth) from Isle du Levant (close to Toulon) to Corsica and back. The total horizontal distance (computed from GPS) is 500 km. The temperature of the whole cruise was documented, and this information (an average temperature of 18 °C), was used in the DGT calculations.

The sampling locations of total dissolved metal concentrations are following: dissolved Cd, Mn, Fe, Ni and Cu concentrations^44^ were measured in the Bay of Villefranche (43.696°N, 7.316°E), which is close to our sampling zone and is continuously flushed with offshore water by the Liguro-provençal current, making the bay oligotrophic. The dissolved Co concentration^45^ is the average of the Western Central Basin which also includes the glider cruise track. The dissolved Cd, Fe, Ni and Cu concentrations^18^ also represent average values of the Western Central Basin.

### Calculation of labilities and dissociation rates of metal complex pools measured in fast (*δ*^g^ = 0.0325 cm) and classic (*δ*^g^ = 0.1125 cm) dgts

The existence of two pools of complexes (one inorganic and the other organic), each one with a proper diffusion coefficient and dissociation rate constant *k*_d_ was assumed. Neglecting mixture effects^66–68^, one can approximate the true *ξ* (average for each of the 2 pools) with the *ξ* computed for the case where there is only one ligand in each of the pools under scrutiny. Assuming excess of ligand, the lability degree depends on the dissociation rate constant, the diffusion coefficient and the thicknesses of the diffusion (*δ*^g^ = 0.0325 cm for the fast DGT and *δ*^g^ = 0.1125 cm for the classical DGT) and of the resin domains (*δ*^r^ = 0.04 cm) as derived in^69^,

12 $$\xi =1-\frac{(1+\varepsilon K^{\prime} )}{\varepsilon K^{\prime} +\frac{{\delta }^{g}}{\sqrt{\frac{{D}_{{\rm{ML}}}}{{k}_{{\rm{d}}}(1+\varepsilon K^{\prime} )}}}\,\coth (\frac{{\delta }^{g}}{\sqrt{\frac{{D}_{{\rm{ML}}}}{{k}_{{\rm{d}}}(1+\varepsilon K^{\prime} )}}})+\frac{{\delta }^{g}}{\sqrt{\frac{{D}_{{\rm{ML}}}}{{k}_{{\rm{d}}}}}}(1+\varepsilon K^{\prime} )\tanh (\frac{{\delta }^{r}}{\sqrt{\frac{{D}_{{\rm{ML}}}}{{k}_{{\rm{d}}}}}})}$$

Where *ξ* is the lability degree of the metal complex (1 is full labile and 0 is totally inert), ε = *D*_ML_/*D*_M_ and *K*′ is the conditional stability constant of the complex (i.e. *K*′ = *c*_ML_/*c*_M_).

## Electronic supplementary material

Supplementary Figure 1

Supplementary Figure 2

Supplementary Table 1

Supplementary Table 2
