# Supplementary material for: In situ measurements of micronutrient dynamics in open seawater show that complex dissociation rates may limit diatom growth
Source: Sci Rep. 2018 Oct 31;8:16125. doi: 10.1038/s41598-018-34465-w (PMC6208410; doi:10.1038/s41598-018-34465-w)

In situ measurements of micronutrient dynamics in open seawater show that complex dissociation rates may limit diatom growth.

Willy Baeyens[1*], Yue Gao[1], William Davison[2], Josep Galceran[3], Martine Leermakers[1], Jaume Puy[3], Pierre-Jean Superville[1,4], Laurent Beguery[5].

Figure S1: Cruise track of the SeaExplorer glider in the Mediterranean Sea from Isle du Levant to Corsica and back.

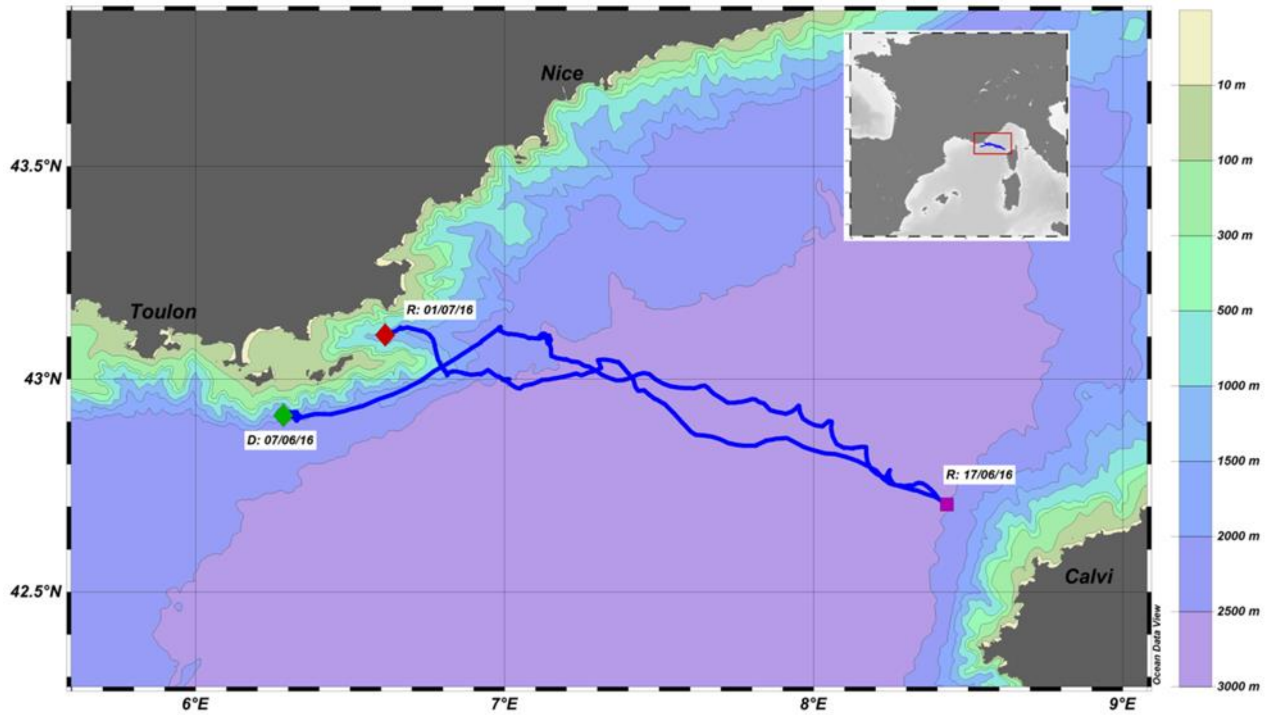

Supplement: Supplementary file 1 — Supplementary Figure 1 [file 41598_2018_34465_MOESM1_ESM.pdf]
