# Supplementary material for: In situ measurements of micronutrient dynamics in open seawater show that complex dissociation rates may limit diatom growth
Source: Sci Rep. 2018 Oct 31;8:16125. doi: 10.1038/s41598-018-34465-w (PMC6208410; doi:10.1038/s41598-018-34465-w)
Supplement: Supplementary file 2 — Supplementary Figure 2 [file 41598_2018_34465_MOESM2_ESM.pdf]

In situ measurements of micronutrient dynamics in open seawater show that complex dissociation rates may limit diatom growth.

Willy Baeyens[1*], Yue Gao[1], William Davison[2], Josep Galceran[3], Martine Leermakers[1], Jaume Puy[3], Pierre-Jean Superville[1,4], Laurent Beguery[5].

Figure S2: Cellular quotas (from Twining et al., 2011; 2015) and seawater concentrations of Fe (from Moore and Braucher, 2007 and Pinedo-Gonzalez et al., 2015), Co (from Pinedo-Gonzalez et al., 2015) and Mn (from Pohl et al., 2011; Butler et al., 2013; Bruland et al., 1994) in various oceanic basins.

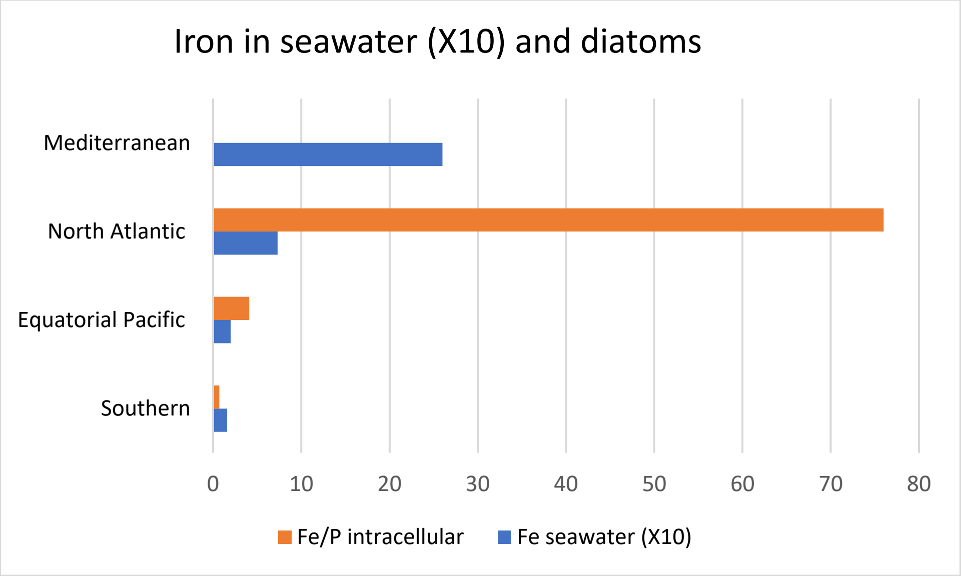

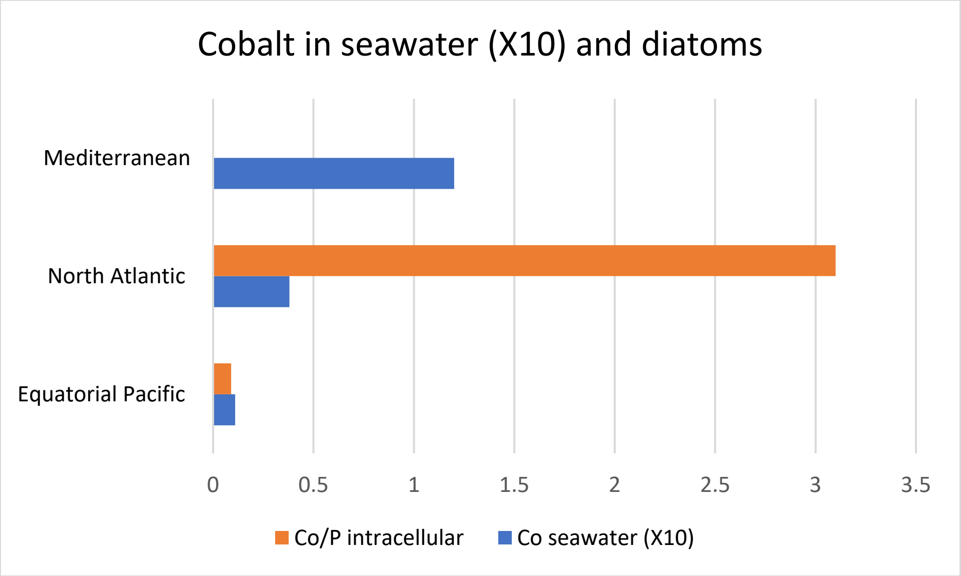

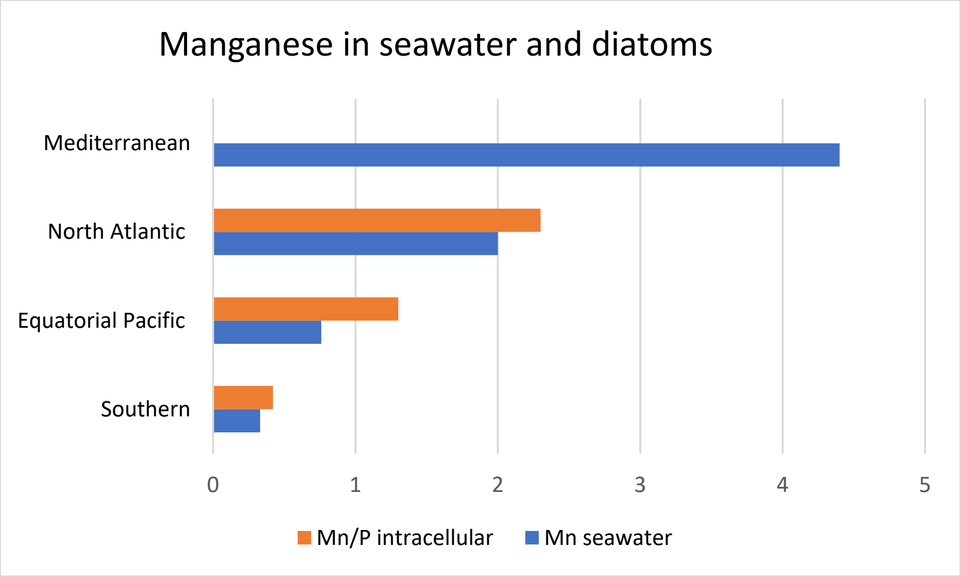

References:

Bruland, K. W., Orians, K. J. & Cowen, J. P. Reactive trace metals in the stratified central North Pacific. *Geochim. Cosmochim. Acta* **58**, 3171-3182 (1994).

Butler, E. C. V. *et al.* Trace metals Cd, Co, Cu, Ni, and Zn in waters of the subantarctic and Polar Frontal Zones south of Tasmania during the 'SAZ-Sense' project. *Mar. Chem.* **148**, 63–76 (2013).

Moore, J. K. & Braucher, O. Observations of dissolved iron concentrations in the World Ocean: implications and constraints for ocean biogeochemical models. *Biogeosci. Discuss.* **4**, 1241-1277 (2007).

Pinedo-Gonzalez, P. *et al*. Surface distribution of dissolved trace metals in the oligotrophic ocean and their influence on phytoplankton biomass and productivity. *Global Biogeochem. Cycles* **29**, 1763-1781 (2015).

Pohl, C. *et al.* Synoptic transects on the distribution of trace elements (Hg, Pb, Cd, Cu, Ni, Zn, Co, Mn, Fe, and Al) in surface waters of the Northern- and Southern East Atlantic. *J. Mar. Syst.* **84**, 28-41 (2011).

Twining, B. S. *et al.* Metal quotas of plankton in the equatorial Pacific Ocean. *Deep Sea Research Part II: Topical Studies in Oceanography* **58**, 325-341 (2011).

Twining, B. S., Rauschenberg, S., Morton, P. L. & Vogt, S. Metal contents of phytoplankton and labile particulate material in the North Atlantic Ocean. *Prog. Oceanog.* **137**, 261-283 (2015).
