# Supplementary material for: In situ measurements of micronutrient dynamics in open seawater show that complex dissociation rates may limit diatom growth
Source: Sci Rep. 2018 Oct 31;8:16125. doi: 10.1038/s41598-018-34465-w (PMC6208410; doi:10.1038/s41598-018-34465-w)
Supplement: Supplementary file 3 — Supplementary Table 1 [file 41598_2018_34465_MOESM3_ESM.pdf]

In situ measurements of micronutrient dynamics in open seawater show that complex dissociation rates may limit diatom growth.

Willy Baeyens[1*], Yue Gao[1], William Davison[2], Josep Galceran[3], Martine Leermakers[1], Jaume Puy[3], Pierre-Jean Superville[1,4], Laurent Beguery[5].

Table S1: c_{DGT} results obtained with classic (0.1125 cm diffusive domain) and fast (0.0325 cm diffusive domain) DGTs and at the head and the tail of the glider. Concentrations in nM.

	Cd	Mn	Co	Ni	Cu	Fe
Head DGT-fast	0.049	0.97	0.058	2.96	0.41	0.47
RSD	6%	17%	8%	9%	9%	33%
Head DGT-classic	0.062	1.69	0.064	3.2	0.94	
RSD	14%	19%	12%	13%	19%	
Tail DGT-classic	0.061	1.25	0.062	3.1	0.73	
RSD	18%	13%	13%	10%	2%	
