# Supplementary material for: In situ measurements of micronutrient dynamics in open seawater show that complex dissociation rates may limit diatom growth
Source: Sci Rep. 2018 Oct 31;8:16125. doi: 10.1038/s41598-018-34465-w (PMC6208410; doi:10.1038/s41598-018-34465-w)
Supplement: Supplementary file 4 — Supplementary Table 2 [file 41598_2018_34465_MOESM4_ESM.pdf]

In situ measurements of micronutrient dynamics in open seawater show that complex dissociation rates may limit diatom growth.

Willy Baeyens[1*], Yue Gao[1], William Davison[2], Josep Galceran[3], Martine Leermakers[1], Jaume Puy[3], Pierre-Jean Superville[1,4], Laurent Beguery[5].

Table S2: Composition of parts of the SeaExplorer. Al, Si and S (in %), other elements (in µg g^{-1}) in (1) Anodized plate and (2) Al plate. Trace metals in polyurethane (PU) material and in leaching solutions. Concentrations in µg g^{-1}.

	Al	Si	S	Cd	Mn	Fe	Co	Ni	Cu	Zn
(1)	75	4.50	18.6	0.11	312	2270	1.87	33	1620	359
(2)	99	0.01	0.02	0.08	291	1940	1.43	24	1460	196

	Cd	Mn	Fe	Co	Ni	Cu
PU	0.0039	4.0	114	0.026	0.388	0.391
HCl 0.1N	0.00092	0.014	0.755	0.001	0.028	0.081
HAc 1M	9.7×10^{-5}	0.011	0.292	0.000	0.013	0.027
HNO_3 1M	0.00058	0.020	0.699	0.002	0.039	0.100
